# Hæmaturia from Umbilical Vein; Constipation

**Published:** 1898-07

**Authors:** O. W. Graham

**Affiliations:** V.S. (O. V. College), Port Perry, Ontario


### HÆMATURIA FROM UMBILICAL VEIN; CONSTIPATION.

By O. W. Graham, V.S. (O. V. College),
PORT PERRY, ONTARIO.

*Subject:* A Clyde colt, eight days old.

*History:* Large and well-nourished, from a dam in good condition; had taken little nourishment for several days.

*Symptoms:* Animal almost continually on the move, but no disposition to lie down; uneasy; movements stiff; frequent attempts to void urine, passing about four ounces of blood each time, after much straining; mucous membrane highly injected; mouth hot and dry; grating of teeth; tongue coated; hurried, panting respiration; small, quick pulse; action of bowels completely arrested; abdomen tender to pressure; abdominal ring enlarged, tense, and painful to manipulation; expression of face dejected.

*Diagnosis:* Hæmaturia from umbilical vein, with constipation.

*Prognosis:* Unfavorable.

*Treatment:* Placed the colt on his back and manipulated the umbilical swelling until a gurgling sound was heard. The colt, on regaining his feet, passed immediately a pint of blood, confirming my conclusion as to the origin of the blood. Placing the colt on the right side, I opened the urachus, which was well closed at the lower end for an animal of that age, but impervious internally. He now bled quite freely. Injected an antiseptic solution of pyoktanin, and inserted a pledget of absorbent cotton soaked in the same. Placed the pledget well up the urachus, stopping hemorrhage completely. Gave as a laxative eight ounces linseed-oil, and every three hours for two days small doses of a mixture of zinc, aromatic spirits of ammonia, belladonna, and ginger, and enemas of warm water. Externally, continual application for twenty-four hours of a woollen blanket wrung out of hot water. On visiting the animal the following day found him urinating freely, without any blood, no movement of the bowels, and not taking any nourishment. The colt continued to remain standing, but was not so uneasy. On removal of the pledget of cotton from the urachus found it closed as before; repacked the opening. Gave four ounces of fluid-extract of cascara sagrada, and continued the stimulant mixture, enemas, and hot cloths externally. Twenty-four hours later a marked improvement was noticeable, the colt taking some nourishment and resting easily, but no action of the bowels. Discontinued the external treatment of hot cloths, and gave four ounces each of castor-oil and linseed-oil, which fifteen hours later was followed by a free movement of the bowels. Removal of the pledget was not followed by any hemorrhage, but a slight leakage of urine and some pus. From this on the colt continued to improve, making a complete recovery. This is the first case of this kind I have noted in fifteen years, though practising in a large breeding-district.

